# Position Dependence of Emission Wavelength of a SiO_2_ Colloidal Photonic-Crystal Laser

**DOI:** 10.3390/polym12040802

**Published:** 2020-04-03

**Authors:** Ting-Hui Chen, Bing-Yau Huang, Chie-Tong Kuo

**Affiliations:** 1 Department of Physics, National Sun Yat-sen University, Kaohsiung 804, Taiwan; final951753@gmail.com (T.-H.C.); flyfishss31@gmail.com (B.-Y.H.); 2 Department of Optometry, Shu-Zen Junior College of Medicine and Management, Kaohsiung 821, Taiwan; 3 Innovation Incubation Center, Shu-Zen Junior College of Medicine and Management, Kaohsiung 821, Taiwan

**Keywords:** wavelength tunable, silica particles, Stöber process, self-assemble, colloidal-crystal laser

## Abstract

In this paper, a wavelength tunable colloidal-crystal laser with monodispersed silica particles was demonstrated. Silica particles were synthesized through the modified Stöber process and self-assembled into the colloidal photonic-crystal structure, which was then used to form the optic cavity of a wavelength tunable laser device. Due to Bragg’s diffraction of the colloidal photonic-crystal and the coffee ring effect, the forbidden energy gap of light varied with different lattice sizes at different positions of the colloidal photonic-crystal. When the pumping pulsed laser irradiated on the gain medium of the sample, the fluorescence was restricted and enhanced by the colloidal photonic-crystal. Lasing emission with a single peak occurred when the energy of the pumping laser exceeded the threshold energy. The threshold energy and the full-width at half-maximum (FWHM) of the proposed laser were 7.63 µJ/pulse and 2.88 nm, respectively. Moreover, the lasing wavelength of the colloidal photonic-crystal laser could be tuned from 604 nm to 594 nm, corresponding to the various positions in the sample due to the coffee ring effect.

## 1. Introduction

The periodic structures of dielectrics have been attractive in recent years in the context of optical communication regimes because of their specific properties, such as the negative index of refraction [1,2], superprism phenomenon [3], and Bragg’s diffraction [4,5]. These phenomena are created by the photonic energy gap in the periodic structure, which is similar to the energy gap of the electron wave in a crystal. Thus, the energy gap of photons is defined as the photonic band gap (PBG), and the periodically dielectric structure is described as a photonic crystal (PhC) [6,7]. The concepts of PhC and PBG were presented by E. Yablonovitch [6] and S. John [7] in 1987, however, one-dimensional PhC with periodically multilayered materials was investigated by J.W.S. Rayleigh in 1888 [8]. Based on the novel properties of PBG, PhC has been applied to communications [3,9], lasers [10,11,12], and displays [13,14] to increase the efficiency of optical devices. For instance, the PhC in a laser device can be used as an optical cavity to increase the efficiency of lasing and decrease the threshold energy of a device via the ability to restrict photons in the PhC [6]. There are several methods for fabricating photonic-crystal lasers, such as lithography [15] and the self-assembly of colloidal particles [4,16,17].

Colloidal crystal (CC) is a high-order structure that is built by monodispersed colloidal particles due to the interactions between colloidal particles (such as static electric force or Van der Waals force) [4,16]. Although CC has only a fake photonic band gap, which means the structure cannot forbid specific wavelengths of light in all directions [4], it has still been investigated by scientists in optical regimes. Compared to the other methods of fabricating PhC, the self-assembling of colloidal particles is a simple and low-cost method to fabricate large scale PhCs. CC lasers have been demonstrated in the past years [4,18,19,20,21].

Previous studies of CC lasers were performed in several types, such as CC immersed in a gain medium [18,22,23] and sandwich structures in which the gain medium is between two CC layers [20,24]. Shkunov et al. presented a tunable lasing device by switching the lattice plane between (111) and (220) in a SiO_2_ CC structure [22]. The wavelength of the lasing emission can be switched between blue and red regions via changing the lattice plane, and the wavelength can be tuned by about 5.0 nm by changing the tilt angle in each region of the CC plate. The PBG of the CC follows the rule of Bragg’s diffraction [4]. When light incidents into a periodically dielectric structure such as a PhC, light with a specific wavelength will be reflected only if it satisfies the conditions of Bragg’s diffraction, which is dependent on the incidence angle, the refractive index, and the lattice constant of PhC. Based on Bragg’s diffraction, the reflected wavelength can be tuned by varying the effective refractive index or lattice constant of the structure.

In this work, the position-dependent emission wavelength of a SiO_2_ CC laser was demonstrated. The lasing could be generated from a dye solution sandwiched by two CC layers. The lasing wavelength could be tuned depending on the position of the sample because of the different lattice constants of CC. The proposed SiO_2_ CC laser exhibited the advantages of having a low threshold and a low cost, as well as wavelength tunability.

## 2. Materials and Methods

Monodispersed silica colloidal particles were prepared by the modified Stöber method [25,26] prior to the fabrication process for the CC. In the fabrication of monodispersed silica colloidal particles, tetraethyl orthosilicate (TEOS, 98%, from ACROS, Geel, Belgium), ammonia (from ECHO, Miaoli, Taiwan), deionized water, and ethanol (from J.T. Baker, Phillipsburg, New Jersey, USA) were mixed and reacted for around 24 h. Afterward, synthesis of the silica particles was completed, and the particles were washed using deionized water and ethanol repeatedly for at least six rounds. The silica colloidal particles were deposited on hydrophilic glass plates and then immersed in a piranha solution for around one hour at 80 °C [17]. The piranha solution was a mixture of sulfuric acid (98 wt%, from ECHO) and hydrogen peroxide aq (ca. 30 %, from ECHO) at a volume ratio of 3:1.

**Figure 1:**
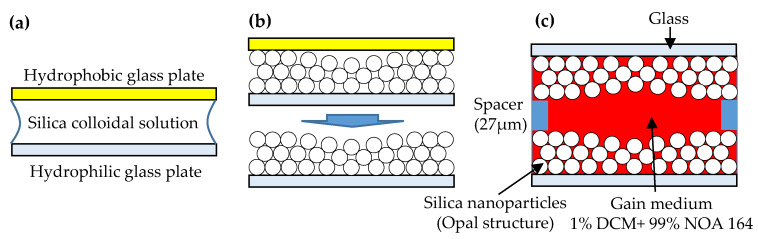
Sample fabrication of the colloidal-crystal laser: (**a**) the silica colloidal solution was placed between the hydrophobic and hydrophilic glass plates; (**b**) the silica colloidal particles were self-assembled into the opal structure; (**c**) two opal plates were combined and injected with the DCM-doped NOA 164 solution to finish the sample fabrication.

The silica colloidal solution was dropped onto a hydrophilic glass plate, and then another hydrophobic glass plate was placed over the silica droplet and glass plate. When the water in the silica colloidal solution evaporated, the silica particles self-assembled into high order arrays as the CC on the glass plate because of the coffee ring effect [27,28], which is a pattern caused by the evaporation of particle-laden liquid. A drop of particle-laden liquid on a substrate will cause a ring-shape composite after evaporation. Due to capillary force, the boundary of particle-laden liquid on the substrates will be maintained. Therefore, an edge-ward flow is produced to bring particles from the middle to the boundary of the particle-laden liquid during evaporation. In this experiment, because a glass plate was covered with the SiO_2_ droplet, the evaporation of the solution occurred first from the edge of the substrate. Due to the edge-ward flow, the concentration of the droplet in the middle decreased during the process. The decrease in concentration resulted in different reflection spectra caused by the different lattice structure of the CC [29]. Two pieces of self-assembled CC plates were assembled into an optical cavity by placing two 27-μm-thick spacers between them. The cavity was then filled with a gain medium, which was composed of 1.0 wt% laser dye, DCM (from Sigma-Aldrich, St. Louis, MO, USA) and 99.0 wt% polymer, NOA 164 (from Norland Products Inc., Cranbury, NJ, USA), as shown in Figure 1.

A silica CC structure was used as the optical cavity in the CC laser for this experiment. The structural integrity of the optical cavity played an important role for generating the lasing of the sample, which could be determined by measuring the reflection spectrum of the CC structure. The wavelength of the reflection spectrum of the CC structure could be described by Bragg’s diffraction, as shown in Equation (1):

*mλ* = 2*ndsinθ*, (1)

where *m* is the order of diffraction, *λ* is the diffraction wavelength, *n* is the effective refractive index of the CC, *d* is the lattice constant, and *θ* is the angle between the incident light and the surface plane. The refractive indices of the silica particles and NOA 164 were 1.45 and 1.64, respectively. Because the CC was assembled as a face-centered cubic (FCC) structure [4,16] and the diffracted surface was (111) plane, the lattice constant of the CC could be calculated by Equation (2):

(2) $$\;d=\sqrt{\frac{2}{3}}a\;\approx \;0.816a,$$

where *a* is the average diameter of the monodispersed silica particles. In the CC laser, the optical cavity formed by the CC structure was used to accumulate the stimulated emission from the DCM-doped NOA 164. The particle size of the silica particles had to be modulated to ensure that the fluorescence of the DCM-doped NOA 164 would be limited in the optical cavity of the CC.

**Figure 2:**
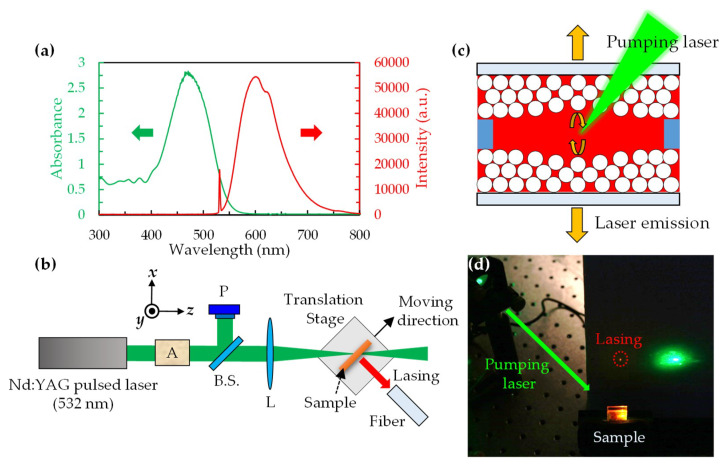
(**a**) Absorption and fluorescence spectra of the DCM-doped NOA 164; (**b**) Experimental setup for measuring the lasing spectrum of the sample at different irradiating position, where A is the attenuator, P is the power meter, B.S. is the beam splitter, and L is the convex lens, respectively; (**c**) Schematic representation of the laser emission from the CC sample; (**d**) Photograph of the CC laser system.

Figure 2a presents the absorption and fluorescence spectra of the DCM-doped NOA 164. The fluorescence spectrum was obtained by pumping the DCM-doped NOA 164 with a 532 nm diode-pumped solid-state (DPSS) laser beam and was received by a fiber-connected spectrometer. The pumping beam was incident into the DCM-doped NOA164 with an incidence angle of 45°. The fiber was set in the normal direction of the cell to receive the fluorescence. The absorption spectrum of the DCM-doped NOA 164 was between 400 nm and 550 nm, while the fluorescence spectrum was at a range of 550 nm to 700 nm. The diagram of the experimental setup for measuring the lasing spectrum at different positions in the sample is shown in Figure 2b. The Nd:YAG pulsed laser (Surelite II, from Continuum, Santa Clara, California, US) with a wavelength of 532 nm and a pulsed width and frequency of 6 ns and 10 Hz, respectively, was chosen as the pumping source in this experiment. An attenuator (A) was placed in front of the pulsed laser and used to adjust the energy of the pumping source being irradiated on the sample. The adjusted pulse laser was then divided into two beams with equal intensities but mutually perpendicular paths by a beam splitter (B.S.). One of the divided beams passed through a convex lens (L) and was further focused on the sample with an incidence angle of 45°, while the other was received by a power meter (P) in order to determine the pumping energy irradiated on the sample. The sample was placed on a precise translation stage to control the pumped location. An optical fiber was used to receive the lasing emission to a spectrometer (USB4000, from Ocean Insight, Largo, Florida, US) at the normal direction of the sample. A schematic representation and photograph of the laser emission from the CC sample are shown in Figure 2c,d, respectively. The laser dye was pumped by the pulsed laser and emitted fluorescence between the CC layers. The fluorescence was reflected by the CC and enhanced until the overall gain exceeded the loss. The lasing was then emitted from the CC via the enhancement of fluorescence collected by the CC.

## 3. Results and Discussion

**Figure 3:**
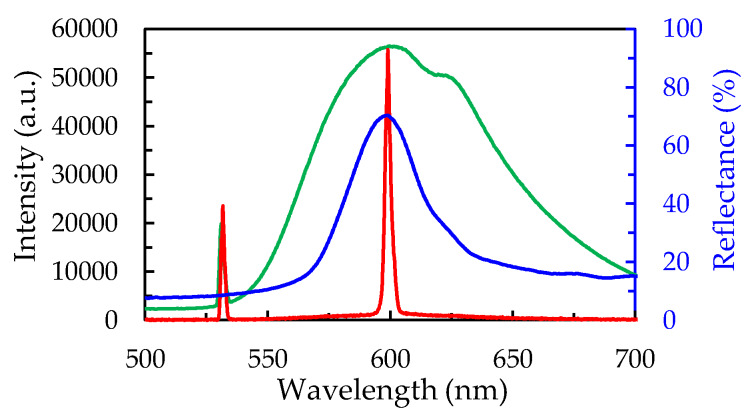
The spectral overlap of the fluorescence (green line), reflection (blue line), and lasing emission (red line) of the SiO_2_ photonic-crystal laser.

Figure 3 displays the spectral overlap of the reflection (blue), fluorescence (green), and lasing (red) of the sample. The reflection spectrum was measured using a white light source (400–800 nm), a reflective fiber, and a spectrometer. The reflective fiber was aligned in the normal direction of the sample to emit and receive the incident and reflected light. The pumping energy of the pulsed laser irradiated on the sample was about 10 µJ for each pulse. The small peaks at 532 nm in the lasing and fluorescence spectra were contributed by the pumping laser. The fluorescence spectrum of the DCM-doped NOA 164 was from about 550 nm to 700 nm under the excitation of the 532 nm green laser, and the peak wavelength was about 600 nm. The lasing occurred at about 600 nm, which corresponded to the peak wavelength of the reflection spectrum of the CC structure and was located on the range of the fluorescence spectrum of the DCM-doped NOA 164. In this laser device, photons were emitted from the DCM-doped NOA 164 and were enhanced by the CC structures. The light of fluorescence was propagated and restricted in the CC structure, resulting in the accumulation of fluorescence in the CC structure. Especially, the highest density of photons existed at the location where the fluorescence and the peak of the reflection spectrum overlapped. Consequently, the stimulated fluorescence at the wavelength of the reflection peak could be greatly accumulated and eventually turned into the lasing emission at the above described location, as shown in Figure 3.

**Figure 4:**
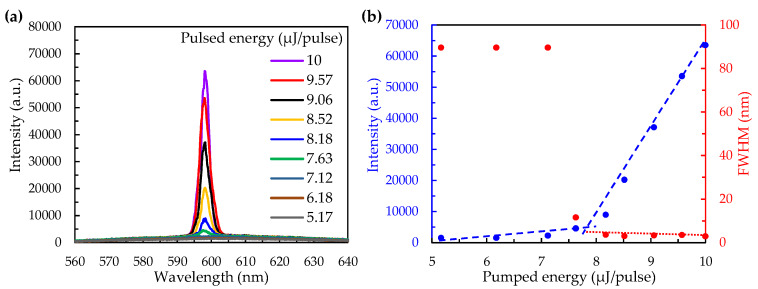
(**a**) The change of the pumping energy on the lasing spectra of the colloidal-crystal laser; (**b**) The pumping energy dependent intensity and FWHM of lasing on the pulsed laser.

The change of the pumping energy on the lasing spectra of the colloidal-crystal laser is shown in Figure 4a. When the pumping energy was below 7.63 μJ/pulse, the lasing could not be observed because the amount of excited electrons in the gain medium was not enough for the population inversion. Most of the stimulated light would be lost by scattering or the re-absorption effect during the accumulation in the optical cavity. However, when the pumping energy was above 7.63 μJ/pulse, the enhancement of the stimulated light led to the population inversion, resulting in the lasing emission. The relation between the pumping energy and the lasing features, including intensity and full-width at half-maximum (FWHM), is shown in Figure 4b. The threshold pumping energy of the laser was about 7.63 μJ/pulse. Below the threshold energy, the fluorescence of the DCM-doped NOA 164 increased with increasing the pumping energy, while the FWHM retains barely defined. When the pumping energy exceeded the threshold, the intensity of the lasing emission rigidly increased and the FWHM decreased rapidly until 2.88 nm, the finally stable value.

**Figure 5:**
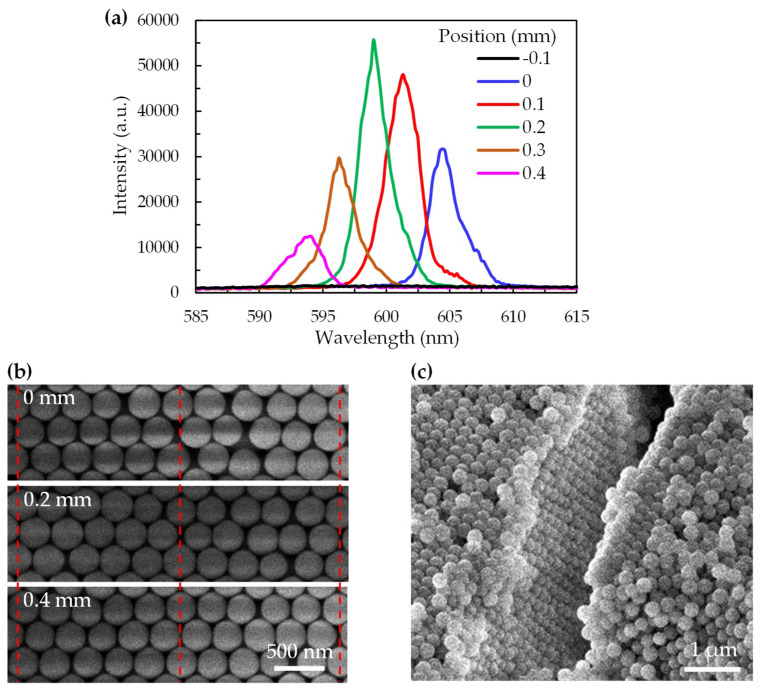
(**a**) The lasing spectra measured at different positions of the sample, in which position of 0 mm is defined as the position where the longest wavelength of lasing occurs; (**b**) The SEM images of the CC layer at position of 0, 0.2, and 0.4 mm; (**c**) Cross-sectional SEM image of the CC.

The lasing spectra measured at different positions of the sample are shown in Figure 5a. The irradiated points, which moved horizontally from the center to the edge of the sample, corresponded to the positions of −0.1 mm to 0.4 mm. The result indicated that no lasing emission was emitted at the position of −0.1 mm. The position of 0 mm in the Figure 5 represents the position of the sample where the longest lasing wavelength occurred, and positions of 0.1–0.4 mm are the displacements from the position of 0 mm in the moving direction of the sample, as shown in Figure 2b. Here, the pumping energy of the pulse laser was set at about 15 μJ/pulse. The wavelength of the lasing emission could be tuned from 604 nm to 594 nm by moving the position from 0 mm to 0.4 mm. Under the excitation with the same pumping energy, the peak intensity of the lasing emission increased slightly and then decreased when the lasing wavelength blue-shifted from 604 nm to 599 nm and from 599 nm to 594 nm, respectively. The tendency of the lasing intensity relied on the photon density, which in turn relied on the enhancement of DCM and the reflective indices on different positions. The blue shift of the lasing spectrum was ascribed to the lattice constants of the CC in the sample having small shrinkage, as shown in Figure 5b, because the variety of the driven force and particle concentrations slowly decreased during the self-assembly process, resulting in the slight change in the photonic band gap of the CC. Figure 5c is a cross-sectional SEM image of the CC and shows the SiO_2_ particles aligning in a typical FCC structure.

**Figure 6:**
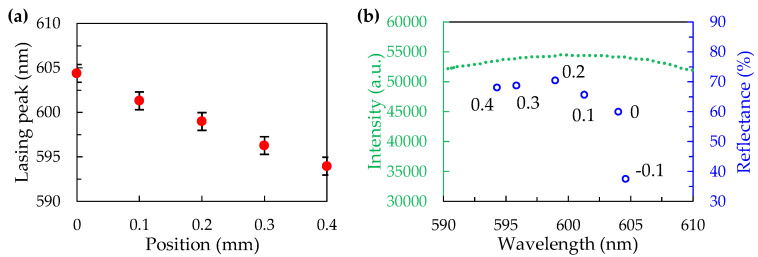
(**a**) The shifted peak wavelength of the lasing spectrum of the sample depending on different positions; (**b**) The peak wavelengths and reflectance of the reflection spectrum of the silica CC filled NOA 164 at different positions along with the fluorescence spectrum of the DCM-doped NOA 164.

In order to realize the relationships among lasing, position, reflectance, and fluorescence, the results shown in Figure 5 were collected, summarized, and replotted, as shown in Figure 6. The dependence of wavelength of the lasing peak on the measuring position, as shown in Figure 6a, exhibited a linear relationship and blue shift while the position was moved from 0 mm to 0.4 mm. The error bars of the peak wavelength at each position were about 2.0 nm, which tested the repeatability and reproducibility of the lasing spectra at different positions in three samples. Figure 6b presents the peak wavelengths and the reflectance at different positions (−0.1–0.4 mm) of the silica CC filled with NOA 164 (blue circles) along with the fluorescence spectrum of the DCM-doped NOA 164 (green dotted line). The peak wavelength of the reflection spectrum decreased from 604 nm to 594 nm and the position increased from 0 mm to 0.4 mm; however, the corresponding peak reflectance first increased to 70% at 0.2 mm and then generally decreased. Since the peak of the fluorescence spectrum was at about 599 mm, the position of 0.2 mm exhibited the highest intensity and lowest threshold lasing emission due to the contributions of the intensive fluorescence and strong reflectance at 599 nm. However, lasing did not occur at the position of −0.1 mm because the silica CC structure had a relatively weak reflectivity of about 38%. The low reflectance implied that the quality factor of the CC structure at this position was not sufficient for the generation of lasing emission.

Furthermore, the peak wavelength of the lasing emission occurred at the peak wavelength of the reflection spectrum overlapping with the fluorescence spectrum. The lasing spectrum shifted depending on the shift of the wavelength of the peak reflectance at different positions. In our experiment, the particle size of the monodispersed silica particles was selected before the sample fabrication, thus the reflection wavelength of the silica CC filled NOA 164 based on Bragg’s diffraction relied on the lattice constant of the CC. The wavelength shift of the reflection spectrum was attributed to the self-assembly of the silica CC. According to the coffee-ring effect [27,28], the monodispersed silica particles were driven to the sides of the substrates by the capillary force. The evaporation rate of the solvent was higher at the edges of the substrates compared to the bulk region inside the sample. Therefore, the silica particles accumulated and self-assembled into an artificial opal structure along the edge of the sample and moved toward the center due to the edge ward flow resulting from the difference between evaporation rates. The concentration of the silica colloidal solution and the edge ward flow decreased because the silica particles were stacked at the side of the substrates, resulting in the difference of the silica CC on the substrates.

The lattice constant (with respect to the position of the CC) could be calculated using Equation (1). First, we considered the first-order diffraction of *m* = 1 and the reflection spectrum of the CC measured in the normal direction; thus, *sinθ* = 1. Then, the effective refractive index of *n* was calculated using the packing factor of the FCC structure and the refractive indices of NOA 164 and SiO_2_. The average diameter of the SiO_2_ colloidal particle was about 240 nm. The peak wavelength of reflection depended on the position shown in Figure 6b, and the lattice constant with respect to the position of 0–0.4 mm was found as 198.6, 199.1, 200.2, 201, and 201.9 nm, respectively.

## 4. Conclusions

The position dependent emission wavelength of a CC laser was demonstrated in this paper. Monodispersed colloidal silica particles were produced by the modified Stöber process and then stacked into the CC structure using self-assembly. The laser dye DCM was doped in the polymer NOA 164 as the gain medium, while the CC structure with a dielectric period was employed as an optical cavity for the fabrication of a CC laser. As the lasing occurred at a wavelength of 599 nm in the normal direction of the sample, the threshold energy and FWHM were about 7.63 µJ/pulse and 2.88 nm, respectively. Furthermore, the lattice constant, reflective wavelength, and lasing wavelength of the CC structure exhibited a small blue-shift when the probe or pumping light moved outward of the sample because of the coffee ring effect, which involved a variety of driving forces and particle concentrations. Under position-dependent lattice constants, the wavelength of the lasing emission could be tuned from 604 nm to 594 nm, which corresponded to different positions from 0 to 0.4 mm.
